# A remotely operated airboat for metal-free, ultraclean water sampling for trace elements in lentic waterbodies: from design and fabrication to operation in the field^☆^

**DOI:** 10.1016/j.mex.2025.103731

**Published:** 2025-11-22

**Authors:** Tommy Noernberg, Taylor Bujaczek, William Shotyk

**Affiliations:** a Department of Renewable Resources, University of Alberta, Edmonton, AB TG6 2R3, Canada; b Bocock Chair for Agriculture and the Environment, Department of Renewable Resources, University of Alberta, Edmonton, AB, T6G 2H1, Canada

**Keywords:** Environmental research vehicle, Remotely operated airboat, Environmental monitoring, Water quality, Tailings pond water, Water sampling

## Abstract

The mitigation of contamination during water sampling remains one of the primary challenges for trace elements research. In shallow water bodies, it is especially important to avoid disturbing the sediments to avoid the introduction of particles and colloids containing trace elements to the water column. Traditional sampling protocols can stir up sediments, and conventional equipment containing metal alloys presents additional sample contamination risks. Here we describe the design, construction, and testing of a metal-free unmanned, remotely operated airboat for sampling lentic freshwater habitats. The SWAMP airboat consists of two pontoons that support a water sampler containing four winches to lower and raise 60 mL bottles to sample from desired depths. The airboat is driven by two motorized propellers and is equipped with Cube Autopilot to stabilize the airboat during sample collection.

• A 3D printer was used to construct plastic components for the airboat, composed of polyethylene terephthalate glycol (PETG), PolyMide™ CoPA nylon, high density polyethylene (HDPE), and polycarbonate (PC) carbon fiber.

• Solenoids were programmed to remotely open and drop weights that open valves on the 60 mL sampling bottle to collect water at specified depths.

• The SWAMP airboat was successfully field-tested at two locations in Alberta, Canada.

## Graphical abstract

**Figure:**
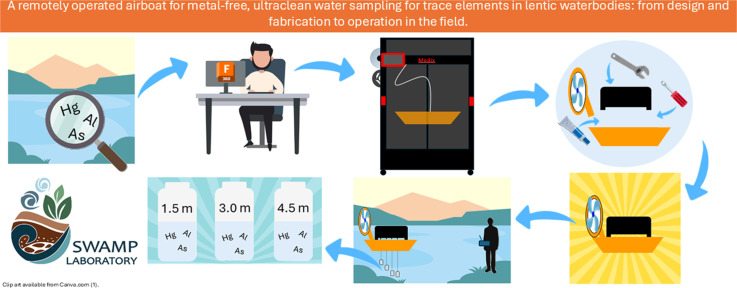

## Specifications table

**Table:** 

**Subject area**	Chemistry
**More specific subject area**	Environmental Chemistry
**Name of your method**	A remotely operated airboat for metal-free, ultraclean water sampling for trace elements in lentic waterbodies: from design and fabrication to operation in the field
**Name and reference of original method**	Not Applicable
**Resource availability**	*Metal Alloys:*
	**316 stainless steel button head hex drive screws**
	https://www.mcmaster.com/products/screws/head-type~rounded/316-stainless-steel-button-head-hex-drive-screws/?s=316+stainless+steel+screws
	*Plastics:*
	**High density polyethylene filament**
	https://www.mcmaster.com/products/rods/plastic-1~/?s=hdpe+rod
	**Polycarbonate carbon fiber filament**
	https://www.amazon.ca/Creality-Filament-Hyper-PLA-CF-Dimensional/dp/B0CP7SFQG9/ref=sr_1_7?crid=36H7NROBX0GY7&dib=eyJ2IjoiMSJ9.CjY46qT42_YN1vqlIs8NljQt9rzvSvMmqs_B2fdzCpRSj8RUeiw0T45H_W-6j36RYJ5B3_4P5JluUhqv6py6Ta19-ugSmgriaT0d_K6Hdi-HXYlL7wY9DC4wr3FCGdd4GT4D03wkZvHHfU0KvmF5CLHRIi_9O3r1Gvx3RXLEMJOVCxt-pQA3W0pa1posrqQbMFXGqG2nIEd6JNO7ZA9J-x6S6d_WSPWtgAalZq7gLLQQhIqHzTtcDBzXQYzBHBic9W2Ij2izQSzcwkyedHCHeebW1OFAaRvFJvD5kDtegeY.YIw4Pkvq9mpNWkokDJFyF-Buuwq5RRMt9ldeKSvmnWo&dib_tag=se&keywords=carbon%2Bfiber%2Bpolycarbonate%2Bfilament&qid=1752087134&sprefix=polycarbonate%2Bcarbon%2Bfi%2Caps%2C136&sr=8–7&th=1
	**Polyethylene terephthalate glycol 3D printer filament**
	https://www.amazon.ca/SUNLU-Filament-Toughness-Filaments-Printing/dp/B0B99PCLYN/ref=sr_1_8?crid=3QWHO6SWIAMU1&keywords=petg%2Bfilament%2B1.75&qid=1701197750&sprefix=petg%2B%2Caps%2C116&sr=8–8&th=1
	**PolyMide™ CoPA nylon**
	https://polymaker.com/product/polymide-copa/
	*Hardware:*
	**AGFRC winch motor**
	https://www.amazon.ca/AGFRC-Waterproof-Digital-Servo-High-Torque/dp/B09133W2B2/ref=sr_1_1?crid=91KS5V23VIOL&dib=eyJ2IjoiMSJ9.UxNZzL3rJW8pCJvZWWnaQRMom5oFIX8hn7w3TacN5OqkR_LZF2bk_kyARiIa_MyABD3CIS8UJiAao13rbvefg0oK_StlthvNXzsOx3zxrzkokOTIV6uK6TuxVlA1L09rDWYufFICe5B7sYr6HUcK5VhJvfCnboYk3t7ozPBZtxwsFOCOYYF71OttvfshSSnoAj-J1Ce3XF9_YCFZ13nVRsB9jfp94DnFYQh7vMWojYH9EuPtVWgPicRFs-9IO_WD7GZVuZTPBVYRLFbvJR7OT1SOIyNs4gRKQS_zyujBEeY.qPUM73gWGWFdf3v2XnjFkyUMWJ6OpKTD89o858t0FHQ&dib_tag=se&keywords=AGFRC+winch+motor&qid=1752087325&sprefix=agfrc+winch+motor%2Caps%2C203&sr=8–1
	**Braided nylon ice fishing line**
	https://www.amazon.ca/Mason-Tip-Up-Fishing-Braided-50TB-30/dp/B0000AUUNL/ref=asc_df_B0000AUUNL/?tag=googleshopc0c-20&linkCode=df0&hvadid=706747096492&hvpos=&hvnetw=g&hvrand=7383935100529709110&hvpone=&hvptwo=&hvqmt=&hvdev=c&hvdvcmdl=&hvlocint=&hvlocphy=9001384&hvtargid=pla-875165936292&psc=1&mcid=17e7c77c4c0e3fcfa0baa99f41264844&gad_source=1
	**Carbon fiber propellers**
	https://store.tmotor.com/product/polish-carbon-fiber-20×6-prop.html?srsltid=AfmBOooqZCyw98XLGcY8BfMvr6LHjxzW7a4Nla4N5P2jk50OIBzrnr8Q
	**TMotor UAV fixed wing aircraft shaft motor for propellers**
	https://store.tmotor.com/product/at7224-fixed-wing-motor.html?srsltid=AfmBOorykgSd1Bfb9It-gG_-fQ4SKtne3nJDNRNKP6FAjZdIlvkyTUQg
	**Carbon fiber tube**
	https://www.amazon.ca/Carbon-Length-Printer-Airplane-Manipulator/dp/B0CX961JCG?th=1
	**Cube Autopilot**
	https://ca.robotshop.com/products/cube-orange-imu-v8?gad_source=1&gclid=Cj0KCQjwiOy1BhDCARIsADGvQnBD5DsWdJGaJj8TMrxB0H_2JqDG9OoeiZWRNNRH1RSkJBLim4KpaC8aAjHiEALw_wcB
	**CubePilot Herelink HD video transmission system V1.1**
	https://ca.robotshop.com/products/herelink-controller-unit-v11?gad_source=1&gclid=CjwKCAiA5eC9BhAuEiwA3CKwQhV4_qtUlMk6YfxhnL7mU02JKTYSQTXICv_-jTUlo8×5LiZ51×1CZBoCK4kQAvD_BwE
	**Dakota** l**ithium 12B 10Ah LiFePO4 battery**
	https://www.cabelas.ca/product/130371/dakota-lithium-12-volt-10-ah-battery
	**Epoxy resin**
	https://www.mcmaster.com/products/epoxy-resins/
	**FLIR thermal camera**
	https://www.itm.com/product/flir–436–0022–00-vue-pro-r-radiometric-thermal-camera-with-9-mm-lens-for-drones?srsltid=AfmBOooTtfHVLmd-J5KXR-_dZcOXUztS5LtCeIGVkp-IjQi8z1CzW12f
	**Foxeer BOX 2 4K HD action FPV aamera supervision HD 155**° **ND filter APP micro HDMI fast charge type-c**
	https://www.foxeer.com/foxeer-box–2–4k-hd-action-fpv-camera-supervison-hd-155-degree-nd-filter-app-micro-hdmi-fast-charge-type-c-g-222
	**FrSKY remote flight pack**
	https://www.frsky-rc.com/product/tandem-x20/
	**FrSKY TD MX receiver**
	https://www.3dxr.co.uk/radio-gear-c33/receivers-c112/frsky-td-mx-receiver-p5518
	**Garmin livescope live sonar and transducer**
	https://www.gpscity.ca/garmin-echomap-94sv-uhd-and-livescope-xr-system-with-gls-10-and-lvs-62-bundle
	**Gremsy gimbal stabilizer**
	https://www.3dxr.co.uk/camera-fpv-c57/gimbals-c214/gremsy-aevo-p5312
	**SIKA Low expansion polyurethane spray foam**
	https://www.amazon.ca/SIKA-Expansion-Polyurethane-All-Season-Application/dp/B0886W3RWK/ref=asc_df_B0886W3RWK?mcid=c98644ca5edb37de95018703280ff087&tag=googleshopc0c-20&linkCode=df0&hvadid=706725384621&hvpos=&hvnetw=g&hvrand=3098161065753967708&hvpone=&hvptwo=&hvqmt=&hvdev=c&hvdvcmdl=&hvlocint=&hvlocphy=1001808&hvtargid=pla-1055716161318&hvocijid=3098161065753967708-B0886W3RWK-&hvexpln=0&gad_source=1&th=1
	**Thermo Scientific™ Nalgene™ wide-mouth lab quality PPCO bottles with closure 60 mL**
	https://www.fishersci.ca/shop/products/nalgene-wide-mouth-lab-quality-ppco-bottles-closure/02893BB?searchHijack=true&searchTerm=02893BB&searchType=RAPID&matchedCatNo=02893BB
	**Ultra-Hard C2 tungsten carbide balls**https://www.mcmaster.com/products/~/material~tungsten-carbide/?s=tungsten+balls
	**Youme Power Li-Poly battery 6500mAh 60C lithium battery**
	https://www.amazon.ca/Youme-Battery-Connector-Airplane-Helicopter/dp/B07DDGY6CY/ref=asc_df_B07DDGY6CY/?tag=googleshopc0c-20&linkCode=df0&hvadid=706724959887&hvpos=&hvnetw=g&hvrand=7188991983693806353&hvpone=&hvptwo=&hvqmt=&hvdev=c&hvdvcmdl=&hvlocint=&hvlocphy=9001286&hvtargid=pla-624466090531&psc=1&mcid=2f26eab43b5d3f4e96baf1dfec913048&gad_source=1
	*Software:*
	**ArduPilot Mission Planner**
	https://ardupilot.org/planner/
	**Ground Control Stations**
	https://ardupilot.org/plane/docs/common-GCS.html
	*3-D Printer:*
	**Modix3D 120Z V4 large format 3D printer kit**
	https://ca.robotshop.com/products/3d-printing-canada-usa-modix3d-120z-v4-large-format-3d-printer-kit?gad_source=1&gad_campaignid=20151185247&gbraid=0AAAAAD_f_xySQ93yIT1Z8NU7nKINu_ix-&gclid=CjwKCAjwprjDBhBTEiwA1m1d0plFkF67TT0VwxJGVSqZpJOIA1jE-bHQ4CGPe1oLbFG3F77uXBme2xoCPtcQAvD_BwE

## Background

Contamination of natural waters by non-essential trace elements (TEs) such As, Cd, Pb, Sb and Tl is a global problem, driven by growth in population, resource extraction and processing, industrialization, urbanization, and, increasingly, war. To determine the impacts of anthropogenic activities on TEs in aquatic ecosystems, their natural abundances and the processes governing their biogeochemical cycles, must be known. Determining the background concentrations of TEs in natural waters often requires sampling in remote and possibly dangerous locations with limited accessibility. Many TEs of concern occur in natural waters at extremely low concentrations, and this presents a number of analytical challenges, but these have largely been overcome using well-established procedures and protocols [[1], [2], [3]]. The first risk of contamination begins with water sampling, and it is essential to use metal-free devices and tools that have been properly cleaned to minimize blank contributions [4]. Regarding polyvalent TEs that occur predominantly in colloidal forms, it is vital to avoid introducing particles and colloids through sediment disturbance while sampling [5]. This is a formidable challenge when collecting surface and subsurface water samples in shallow bodies of open water such as natural and constructed wetlands and ponds, including tailings ponds, where unconsolidated sediments can be very easily disturbed [6]. Pumping water using peristaltic pumps may have similar impacts [7]. Introducing particles into the water sample is not fatal, as these are removed by filtering using a 0.45 µm membrane filter. Introducing colloids into the water sample, however, is fatal, in that these are not removed during this filtration step: measured concentrations of TEs such as Al, Cr, Cu, Pb, *Sc*, Tl, and V can easily be inflated by 10 to 1000 times, compared to undisturbed water samples [5].

Traditional water column sampling methods pose several additional risks for contamination. For example, conventional water sampling devices such as the Van Dorn water sampler composed of metals or metal alloys create the risk of direct sample contamination [8]. Unmanned aerial vehicles (UAVs) have been used to collect water samples in remote regions using an onboard peristaltic pump with all plastic components [9]. However, with UAVs it is imperative that the method of deployment used does not create new issues with sampling accuracy [7]. For instance, UAVs that land on the water surface may not be designed to control drift, making it far more difficult to accurately collect samples from specific locations (personal observation) and jeopardizing the fidelity of repeated sampling. Additionally, aerial drones often require special permission for deployment at some sites, such as those with nearby airports or heliports; further, they require additional effort regarding operator training, licensing and insurance, creating additional logistical difficulties [7].

Unmanned surface vehicles known as airboats are being used more frequently in freshwater habitats for an array of research [6] and enhance the safety of field personnel in remote or dangerous locations. Airboats are flat-bottomed vessels propelled by onboard propellers that can cruise in shallow waters with minimal disturbance to the water column [10]. Such airboats have been documented to record in-situ water quality parameters by lowering sensors from the vessel to the water [6]. To date, however, we know of no published reports to collect water samples at discrete depths. Designing an airboat that is capable of collecting water column samples at desired depths would eliminate some of the considerable challenges associated with an aerial drone, namely the logistical aspects of permitting and licensing. Materials that are used in sampling equipment, even some plastics, can pose sampling contamination risks due to abrasion or leaching [4], so material consideration is essential. Constructing the airboat using metal-free materials would have the advantage of reducing the risk of contamination by TEs. This is particularly important for the natural resource sector, including fossil fuels (coal, gas and oil), ferrous and non-ferrous open pit mining, and even aggregate extraction and washing: these industries have associated tailing ponds and post-closure pit lakes which require water quality monitoring for their water use management plans, wastewater treatment, and plans for site closure [8]. The goals of this study were to

• Describe the design, components, and operation of an unmanned airboat, constructed of metal-free materials and remotely operated using autopilot technology.
• Investigate the capability of the airboat by testing it in a shallow lentic waterbody and sampling water at desired depths.

## Method details

### Design

The SWAMP airboat was designed using Fusion 360 drawing software and constructed using a Modix 3-D printer. The Modix 3-D printer was first built using a do-it-yourself printer kit (Fig. S7). The plastic polymer filaments used to print all the plastic components include polyethylene terephthalate glycol (PETG), high density polyethylene (HDPE), polycarbonate (PC) carbon fiber, and PolyMide™ CoPA nylon (copolymer of Nylon 6 and Nylon 6.6; [11]). Plastic components were secured together using epoxy resin, but no epoxy resin was used for any of the materials used to collect the water samples. Plastic components that needed to be secured with screws were 3-D printed with threads for seamless configuration. The screws were composed of 316 stainless steel (316 SS) as this alloy does not contribute detectable Pb contamination, as reported when ancient Greenland ice samples were processed using 316 SS chisels [12], and for pristine groundwater sampling from a 316 SS well [13].

### Components

**Fig. 1:**
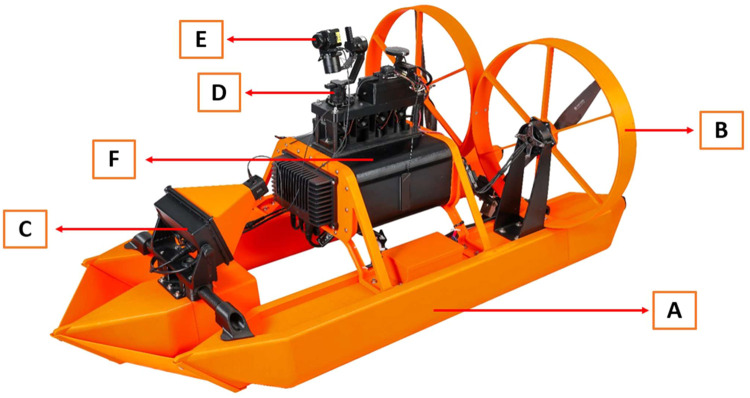
SWAMP airboat featuring the (A) pontoon design with (B) the protectors encasing the propellers, (C) the Garmin Livescope, (D) the Gremsy Gimbal stabilizer, (E) FLIR thermal camera, and (F) the black winch water sampler to deploy 60 mL PP bottles to desired depths.

**Fig. 2:**
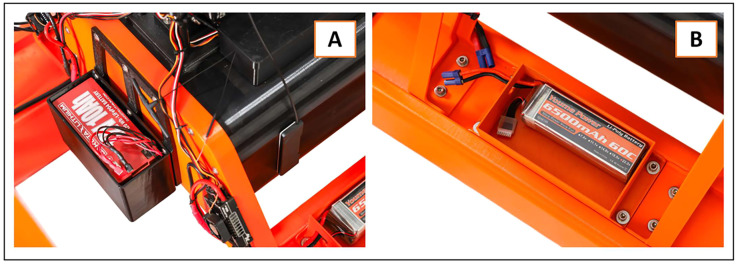
Batteries encased in protective boxes that power the (A) black winch water sampler and (B) motors for the propellers.

The SWAMP airboat has several components that are completely novel coupled with commercially available technologies. The structure of the airboat features two hollow PETG pontoons filled with polyurethane spray foam to allow the airboat to float and navigate smoothly through the water (Fig. 1a). PETG was chosen because it is metal-free and strong, yet has some flexibility to minimize the risk of the pontoons cracking during operation of the airboat. The airboat is powered by 3 batteries: this is ideal because gasoline and other hydrocarbon fuels are often not permitted for use at oil and gas sites. A battery box rests on each pontoon, and the lid allows access to a lithium battery (Fig. 2b). These two batteries are each connected to motors that each power a metal free, carbon fiber propeller. The propellers rest on black PolyMide™ CoPA nylon stands that are secured to the stern with 316 SS screws (Fig. S2). The PolyMide™ CoPA nylon was selected as the propeller stands because this is metal-free, but also because it can withstand the vibration of the rotating propellers, thereby reducing the chance for any cracking of the plastic. Each propeller is also encased in orange circular protectors in PETG for its strength and flexibility (Fig. 1b). The propellers limit water column mixing because they operate above the water surface, as opposed to a conventional boat motor.

**Fig. 3:**
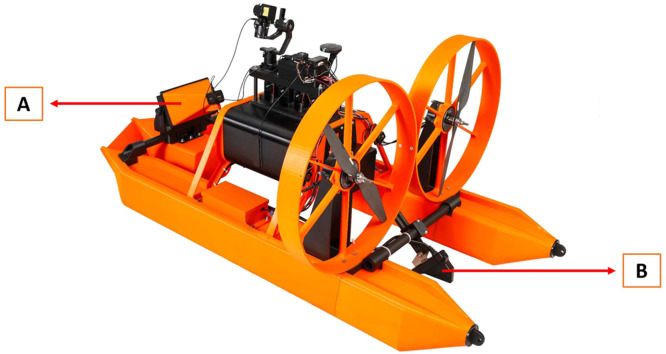
Rear view of SWAMP airboat depicting the (A) Garmin Livescope and (B) transducer.

The SWAMP airboat is equipped with Garmin Livescope, which uses sonar to map the depth of the water body and maps any obstacles found there (Fig. 1; Fig S3). This allows the operator to choose which depth to deploy the sampling bottles and prevents bottles from touching the sediment. It also allows viewing of obstacles in the water, such as debris or vegetation, so the airboat can avoid these areas. Sonar is optimal for local path mapping because it has high-speed processing capability to provide feedback in real time to reduce the chance of collision with underwater objects [10]. The Garmin Livescope is at the bow, and rests on a lightweight yet strong carbon fiber tube that spans the two pontoons (Fig. 3a). At the stern, the Garmin Livescope transducer also rests on a carbon fiber tube that can be lowered into the water manually to transmit the sonar (Fig. 3b). This mapped surface can be viewed using the CubePilot Herelink HD Video Transmission System remote control (Fig. 5; Fig S8), where a Foxeer BOX 2 camera is mounted on the Garmin sonar cover to provide video feedback to the remote control (Fig. S3).

**Fig. 4:**
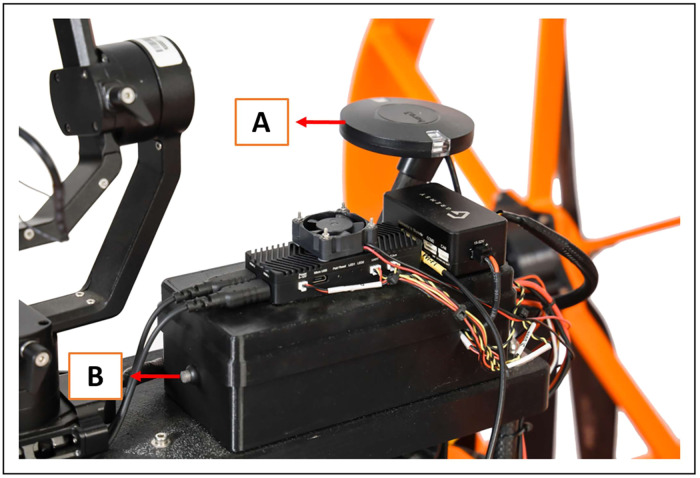
(A) Cube autopilot technology that stabilizes the SWAMP airboat via GPS satellite during sample collection with the (B) emergency safety button.

Cube Autopilot is also installed to limit drifting of the airboat and allows the operator to remotely stabilize the airboat at a specific location (known as Loiter) while sampling (Fig. 4). Locations are verified using GPS technology, and navigation is controlled from the shoreline using the Herelink remote control. The SWAMP airboat also features a FLIR thermal camera mounted on a Gremsy Gimbal stabilizer, which provides a 360° view operated using the Herelink remote control (Fig. 1d; Fig. 1e). The FLIR thermal camera detects changes in water temperature so that any groundwater inputs or springs can be identified and sampled if desired.

The water sampler is located centrally between the two pontoons and elevated above the water by a PETG stand (Fig. 1f). A lithium battery in a third battery box powers the water sampler (Fig. 2a). The water sampler is equipped with four programmable winch motors each with 25 kg towing capability (Fig. S5). Each winch is equipped with approximately 10 m of braided nylon ice fishing line and is tied to a water sample collector (Fig. S5). The winch can be equipped with any length of line, and nylon fishing line is ideal because the line doesn’t curl and is durable.

**Fig. 5:**
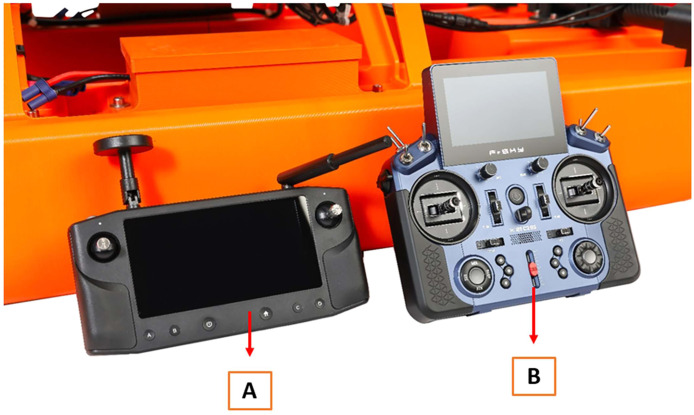
(A) Cube Autopilot Herelink remote control and (B) FrSKY remote control that operates a combination of functions for the SWAMP airboat.

**Fig. 6:**
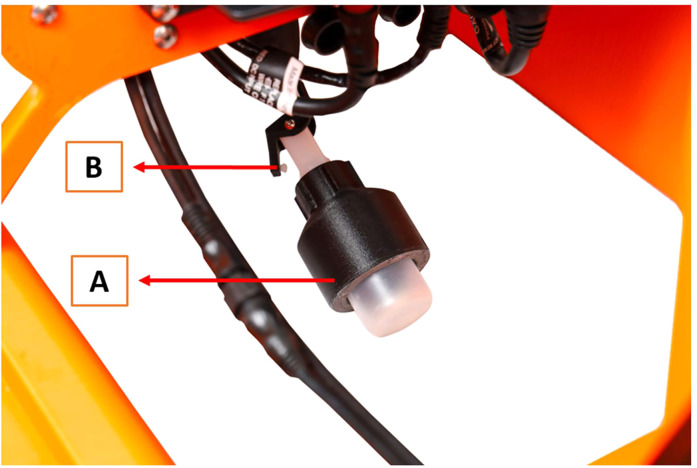
60 mL PP bottle loaded into a (A) PC carbon fiber tungsten ball filled water sample collector featuring the (B) valve opened from the HDPE rod.

The water sample collector is a hollow cylindrical structure consisting of PC carbon that is filled with small tungsten carbide balls (Fig. 6a). The dense tungsten balls allow the water sample collector (containing an empty sampling bottle) to be lowered through the water column. The water sample collector is lowered using a separate FrSKY X205 remote control (Fig. 5b), and the depth of the bottle in the water column can be viewed on the Herelink remote control (Fig. S8). The water sample collector has one opening to load a 60 mL polypropylene (PP) bottle and the opposite end is internally threaded allowing the bottle to be screwed inside the water sample collector (Fig. 6; Video_3). To open the bottle and collect a water sample, the water sample collector is equipped with a small hole that is opened using the FrSKY remote control by releasing a teardrop weight from the solenoid, similar to the operation of a Van Dorn water sampler [8]. The hole is manually sealed in a white HDPE rod before lowering a bottle through the water column: this prevents water from flowing into the bottle while it is being lowered (Fig. 6b; Video_2). The teardrop weight is hooked onto a solenoid that can be released using the FrSKY remote control, and the small weight then slides down the nylon line to the bottle’s release valve (Fig. S5b; Video_2). Water flows into the bottle and the FrSKY remote control raises the bottle to the surface and back into the water sampler. When designing the valve, empty bottles were lowered and raised out of a reservoir filled with water and examined to ensure there was no leakage or opportunity for cross-contamination. As there are four winches each with their own water sample collector, four water samples can be collected during one mission. If greater water volume is required, the operator could take all four samples at the same depth and combine them all to create a bulk sample (240 mL).

After testing many kinds of plastic water sampling bottles, PP bottles were deemed most appropriate for our TE research in pristine surface and groundwaters [13]. A 60 mL bottle collects sufficient volume to measure total (unfiltered, acidified), dissolved (< 0.45 µm, filtered, acidified), colloidal (1 kDa to 0.45 µm, filtered, unacidified) and truly-dissolved (< 1 kDa respectively, filtered, unacidified) concentrations of a wide range of TEs. These samples can all be analyzed at the metal-free, ultraclean SWAMP Lab facility using an inductively coupled plasma mass spectrometer (ICP-MS; iCAP TQ, Thermo Fisher Scientific) and an asymmetric flow-field flow fractionation system (AF4; Postnova Analytics, Salt Lake City, Utah, USA) coupled to a second ICP-MS (iCAP RQ, Thermo Fisher Scientific).

### Operation

#### Pre-Operational safety checks

Prior to deploying the SWAMP airboat into a waterbody, the operator must perform pre-operational checks to ensure safe working conditions for the boat and operator, but also to consider any possible impacts on the environment (Table S1). A summary of these checks and subsequent actions is available in the supplementary information (Table S1). The field site must have an accessible shoreline as the SWAMP airboat is large (66×105×171 cm) and requires two workers to place it in the water. Because it is battery-powered, the operator must avoid rain and splashing to avoid the risk of electrical damage, and the weather must be ≥ 5 °C, for optimal battery efficiency. Operation during high winds (≥ 30 km/h) should be avoided, as this can impact steering and increase the risk of damage. Before sampling with the airboat, the batteries and remote controls should be fully charged. When the SWAMP airboat is placed on the shoreline and powering up, GPS satellites will determine that as the “Home” location. If the battery voltage for the airboat drops under minimum settings or connection to the Herelink remote control is lost, the boat will automatically return to “Home” as a backup safety feature. At this time, both remote controls can be powered on, and all three batteries can be hooked up to the motors and the water sampler. From this point forward, it is imperative to keep hands away from the propellers to avoid injury. Once the power is on, the Cube Autopilot will automatically connect to GPS satellites, and the FLIR thermal camera will also turn on. The Foxeer camera displaying the sonar is turned on and an indicator light will illuminate from blue to green. The SWAMP airboat is also equipped with a safety button located on the Cube Autopilot, where if activated the circuits are tripped in the event of an emergency (Fig. 4b).

To create a mission, waypoints can be created on a mission planner, such as ArduPilot Rover, and uploaded to the Herelink remote control. Ground Control Stations software from ArduPilot allows the operator to configure the settings (such as speed) of the Cube Autopilot system to optimize control of the airboat. Alternatively, waypoints can be selected on the Herelink touchscreen remote control during a mission (Fig. S8).

The water sampler is stored with empty 60 mL bottles that are clean but will not be used for sampling: in the field, these are replaced with acid-cleaned transfer bottles while ensuring the valve is closed (Video_3; [13]). All handling of water samples for TEs follows the clean sampling and handling protocols noted in previous publications [13]. Two workers can gently push the SWAMP airboat into the water, and a test drive is performed to confirm safe operation. To turn left, the left joystick on the Herelink remote control is moved forward, and the left motor rotates according to the pre-set speed configurations in Ground Control Stations software (Video_1). The more aggressive the turn, the faster the propeller will spin to compensate (Video_1). The same test is performed on the right motor and propeller. The test drive confirms manual navigation, Loiter, and Return to Home functions. However, automatic navigation and Return to Home functions are not vital to the airboat operation and are optional safety features. Once proper safety checks have been confirmed (Table S1), the operator can proceed with sample collection.

#### Sample collection

The SWAMP airboat starts at Home and proceeds to the selected waypoint. Ideally, the SWAMP airboat should be facing the wind, as the airboat can make effortless adjustments from any drift as needed during sample collection. After reaching the waypoint and the airboat is stable, the operator selects “Loiter” on the Herelink remote control and stabilizes the airboat at a fixed location within a 2 m radius. If the SWAMP airboat drifts, the Cube Autopilot will automatically make the adjustment to stay within the 2 m radius while sampling. The total depth can be viewed which makes it possible to decide the sampling depths (Fig. S8). The water sample collector is lowered using the FrSKY remote control, and the sonar on the Herelink remote control detects the depth of the descending bottle: this allows the operator to know when to stop lowering the bottle. After the desired depth is reached, a switch is activated on the FrSKY remote control that retracts the solenoid holding the small weight at the water sampler. Next, the weight falls down the line holding the deployed water sample collector, and the valve on the water sample collector is opened to allow water to be collected by the transfer bottle (Video_2). The operator raises and lowers the water sample collector slightly to allow any trapped air to escape the transfer bottle to prevent incomplete filling. The operator raises the water sample collector until it disappears from the sonar feedback and then waits 10 seconds for the bottle to enter back into the water sampler housing (Fig. S6). The operator can now proceed to collect the next water sample. When all samples are collected, the operator can manually navigate the SWAMP airboat back to the launch point or select “Return to Home” on the touchscreen Herelink remote control to automatically return the SWAMP airboat back to the launch point.

Once back on the shoreline, the transfer bottles in the water sample collector are carefully removed while wearing clean lab clothing (e.g. hair net, polyethylene gloves). New transfer bottles are screwed onto the water sample collectors for the next round of water sampling.

The water samples are separated into four different treatments for TE analysis: 1) total concentrations (unfiltered, acidified); 2) dissolved concentrations (< 0.45 µm, filtered, acidified); 3) TEs in the colloidal fraction, ca. 1 kDa to 0.45 µm (filtered, unacidified); and 4) the truly-dissolved fraction, ca. < 1 kDa (filtered, unacidified). In the laboratory, the first two treatments are analyzed using the ICP-MS and the second two using AF4-ICP-MS.

## Method validation

The SWAMP airboat was field tested on two occasions. The first field test took place in June of 2024 at Long Lake, AB (Table S2) to confirm navigation capabilities, Loiter function, and lowering and retrieving of full sample bottles. The SWAMP airboat navigation performed as expected, and the Loiter functioned effectively. However, the operator found that the initial Loiter radius of 5 m was too wide and would need to be narrowed using the Ground Control Stations software. The sonar was also not displayed properly on the Herelink remote control and required further software troubleshooting. After the field trip, the software on the Herelink remote control was successfully modified and the radius of the loiter was adjusted to 2 m instead of 5.

After these modifications were made, a second field test was initially set to take place at Little McLeod Lake, AB in October 2024. However, there were whitecaps on the lake and the wind speed (31.3 km/h) exceeded the safety guideline for safe airboat operation. Field testing proceeded instead at Star Lake, AB where the wind speed was suitable: 1.4 km/h to 10.8 km/h (Table S1). Details regarding the duration of pre-operational safety checks, cruising to the sampling site, and sample collections were recorded during this field test (Table 1). Pre-operational safety checks were performed at the Home location on the shoreline of Star Lake and were passed (Table S2). The SWAMP airboat was deployed from Home to Site 1, approximately 58 m away (Fig. S1; Table S2; [14]). The operator successfully collected three water samples at Site 1, using acid-cleaned transfer bottles at depths of 1.5, 3.0, and 4.5 m. This program was repeated two more times for a total of three missions.

**Table 1:** Descriptive statistics for water sample collection duration (seconds) at three different depths, three different sample collections, and cruising from Home to Site 1 by the SWAMP airboat at Star Lake, AB.

Parameter	Mean	n	SD	SE	Range	Median	Min-Max
Depth 1.5 (seconds)	87.3	3	15.9	9.17	28.0	96.0	(69.0–97.0)
Depth 3.0 (seconds)	111	3	18.6	10.7	37.0	109	(93.0–130)
Depth 4.5 (seconds)	253	3	185	107	367	222	(85.0–452)
Sample Collection 1 (seconds)	96.7	3	12.0	6.93	24.0	96.0	(85.0–109)
Sample Collection 2 (seconds)	140	3	77.0	44.4	153	130	(69.0–222)
Sample Collection 3 (seconds)	214	3	206	119	359	97.0	(93.0–452)
Cruising to Site 1 (seconds)	91.0	3	45.1	26.1	81.0	68.0	(62.0–143)

Sampling at depths of 1.5, 3.0 and 4.5 m ranged from 69.0–97.0, 93.0–130, and 85.0–452 s respectively. The range for sampling at 4.5 m was greater (range = 367 s; Table 1). The operators realized during the third sample collection at 4.5 m that the water sample collector line was caught on the back of the boat and was not reeled in properly. Consequentially, the weight was not released from the water sampler to open the water sample collector, which likely contributed to the longer sampling time. In this situation, bottles were changed out to avoid possible cross contamination, and so this required a few extra minutes (Table 1). The mean sample collection duration increased with each subsequent trip (Table 1). The mean duration (seconds) for the third sample collection (214 ± 119 s, *n* = 3) appears to be 2.2 times greater than the first sample collection (96.7 ± 12.0 s, *n* = 3), although additional field trials would be needed to perform statistical analyses to validate this observation. As already mentioned, this was likely due to the water sample collector not being reeled in properly prior to SWAMP airboat deployment. The total time for the SWAMP airboat to perform three missions at three sampling depths, including changing out bottles and the operational safety checks, was 1 hour and 40 min. Additional deployment of the airboat would likely see enhanced consistency and a reduction in the total sampling time.

### Performance

The SWAMP airboat performed as expected and presents a number of advantages for water sampling in shallow, open waterbodies for TEs. The main advantage of the airboat is that it avoids disturbing the sediments which otherwise could create sampling artefacts by contributing TE-containing colloids to the water column. Second, the propellers eliminate the chance of the airboat becoming entangled with weeds which often occur in shallow bodies of open water [10]. Third, because the airboat is operated remotely, it is ideal for sampling in dangerous locations such as tailing pond waters which may be acidic, corrosive, contain toxic constituents, or release gases that are noxious or dangerous [8]; this presents an additional advantage, as it would avoid permitting and licensing logistics typically associated with UAV and conventional water sampling [10]. Further, the SWAMP airboat is capable of environmental mapping with the sonar technology, lending itself to many possible geotechnical applications such as monitoring bank and dyke stability. The SWAMP airboat is also capable of making automatic adjustments using the Loiter function to remain stabilized at the desired location without drifting. The SWAMP airboat would be ideal for sampling water at shallow depths in natural systems such as wetlands [10], as well as slow moving streams and creeks, and large and small lakes where access by watercraft or canoe may be difficult. The SWAMP airboat demonstrated that it can operate in cold and warm temperatures, so it should find applications globally when waters are ice-free. Finally, we note that the SWAMP airboat was designed specifically to support our research on TEs, but it could also be used for basic water quality mapping and monitoring e.g. dissolved oxygen, electrical conductivity, pH [6], and for collecting water samples to be analyzed for many other chemical constituents including major cations and anions [8], dissolved organic carbon [15], organic contaminants from hydrocarbon extraction and processing [16], and contaminants from military applications [10], to name just a few. We note that sampling pristine surface waters in remote regions of northern Canada probably represent limited risk of cross contamination between samples. However, in regard to sampling tailings ponds from resource extraction and processing, whether they contain hydrocarbons, or heavy metals themselves could pose additional contamination risks. To mitigate against this, spare water sample collectors should be kept on hand and changed at reasonable intervals.

## Limitations

While the field test of the SWAMP airboat was successful, there are some limitations. First, while the airboat only needs one person to operate missions on the water, a minimum crew of two individuals are required to unload the SWAMP airboat (∼20 kg) to the launch point due to its size and weight. This not necessarily a disadvantage, however, since working alone is usually not permitted for field research. But the large size of the airboat may restrict sampling to areas with accessible shorelines, as sites with steep terrain would not be a viable option to deploy this equipment; this disadvantage applies to other approaches e.g. using a canoe for surface water sampling. Other field conditions that impact SWAMP airboat operation and sampling include cold temperatures (≤ 5 °C). The SWAMP airboat was field tested in late October, and the air temperature when sampling started was 7 °C. After about an hour and twenty minutes, the air temperature dropped to ∼5 °C and the FLIR thermal camera lost power. Consequently, the SWAMP airboat cannot be used to sample more than an hour when the air temperature is ≤ 5 °C as it impacts battery efficiency. Once again, however, this limitation would be the case with other equipment powered by batteries. Extra batteries and a charger should be kept onsite to allow batteries to be easily changed.

The volume of water collected in each bottle is sufficient for our TE research, but for other studies of the chemical, biological, or physical characterization of water, larger samples may be needed. Specifically, the SWAMP airboat is currently capable of collecting 60 mL of water in a single bottle, whereas some unmanned aerial vehicles collected samples as large as ∼1.75 L [8]. In future, water sample collectors could be designed to accommodate larger bottles, e.g. using PC carbon and tungsten balls. However, the mass of water collected must be compatible with the hoisting capabilities of the winch. Alternatively, the operator can collect four water samples at a single site at the same depth and combine all the water to create a composite sample. This increases the sampling capability from 60 mL to 240 mL without any structural modifications.

Lastly, a small drawback exists related to the structural design and remote operation. The SWAMP airboat is slightly front heavy, and this is particularly noted when the airboat is coasting through the water, as the bow descends slightly against the water surface. This shortcoming is small but it is advisable for future designs to shift the water column sampler containing the winches towards the stern a few centimeters to compensate for this (Fig. 1). Additionally, the operator must switch back and forth between the Herelink and Freefly remote controls when navigating, viewing the sonar, and taking water samples (Fig. 5). During the time of construction, there was no single remote control that could be configured to broadcast the sonar, navigate the airboat, and lower and retrieve bottles. Currently, newer remote control models are available that could be configured to enable all functions on a single device for effortless operation. Overall, these shortcomings associated with the SWAMP airboat do not impede its operation and is certainly able to perform water sampling for TE research as intended.

## Ethics statements

This material is the author’s own original work and is truthful and complete. The results are appropriately placed in the context of prior and existing research. All authors have been personally and actively involved in substantial work leading to the paper and will take responsibility for its content. The paper properly credits the meaningful contributions of co-authors and co-researchers. All sources used are properly disclosed using correct citation.

## Supplementary material *and/or* additional information [OPTIONAL]

See Supplementary Information for more details.

## CRediT authorship contribution statement

**Tommy Noernberg:** Conceptualization, Methodology, Data curation, Formal analysis, Investigation, Resources, Software, Visualization, Writing – review & editing, Validation. **Taylor Bujaczek:** Writing – original draft, Writing – review & editing. **William Shotyk:** Supervision, Writing – review & editing, Project administration, Funding acquisition.

## Declaration of competing interest

The authors declare that they have no known competing financial interests or personal relationships that could have appeared to influence the work reported in this paper.

## Data Availability

Data will be made available on request.

## Appendix Supplementary materials

Image, application 1

Image, application 2

Image, video 3

Image, video 4

Image, video 5
