# Supplementary material for: A remotely operated airboat for metal-free, ultraclean water sampling for trace elements in lentic waterbodies: from design and fabrication to operation in the field
Source: MethodsX. 2025 Nov 22;15:103731. doi: 10.1016/j.mex.2025.103731 (PMC12720301; doi:10.1016/j.mex.2025.103731)
Supplement: Supplementary file 1 [file mmc1.docx]

***Supplementary Information for***

**A remotely operated vehicle for metal-free, ultraclean sampling of lentic waterbodies for trace elements: from design and fabrication to operation in the field**

**Tommy Noernberg^1^, Taylor Bujaczek^1^, and William Shotyk^2^**

^1^ Department of Renewable Resources, University of Alberta, Edmonton, Canada

^2^Bocock Chair for Agriculture and the Environment, Department of Renewable Resources, University of Alberta, Edmonton, Canada

† Corresponding author: William Shotyk ([shotyk@ualberta.ca](file:///C:\Users\shotyk\AppData\Local\Microsoft\Windows\Temporary%20Internet%20Files\Content.Outlook\W7N38TUY\shotyk@ualberta.ca))

Department of Renewable Resources, University of Alberta, 348B South Academic Building, Edmonton, Alberta CANADA T6G 2H. Tel.: 780-492-7155 Fax: 780-492-4323

REVISED VERSION For submission to MethodsX

Contents

***List of Figures***

**Figure S1:** SWAMP airboat Home and Site 1 locations for airboat field testing at Star Lake, AB on Oct. 24, 2024.

**Figure S2:** SWAMP airboat propellers resting on black 3-D printed PolyMide^TM^ CoPA nylon stands.

**Figure S3**: (A) Garmin Livescope with (B) protective covering that uses sonar mapping to determine ideal sampling locations in a lentic waterbody. A (C) Foxeer BOX 2 camera mounted on the covering feeds Garmin Livescope sonar video back to the Herelink remote.

**Figure S4:** The FLIR thermal camera mounted on a Gremsy Gimbal stabilizer provides a 360° view of the site for the operator to view obstacles and map groundwater inputs into the water.

**Figure S5:** (A) Four AGFRC programmable motors (winches) that raise and lower 60 mL sampling bottles to desired depths. (B) The teardrop PC carbon weights are released from a solenoid released by the operator.

**Figure S6:** The SWAMP airboat displaying the ventral view of the black water sampler with four bottles mounted inside ready for deployment.

**Figure S7:** Modix 3D printer used to construct all plastic components for the SWAMP airboat.

**Figure S8:** Herelink remote demonstrating the (A) y-axis with descending depth (ft), (B) sonar imagery cast by the Garmin Livescope, and (C) display of speed (km/h), flight time (minutes), voltage (v) remaining on batteries, and the distance to Home (m).

***List of Tables***

**Table S1:** Pre-operational safety checks, mission planning, and test drive steps and actions prior to safe operation and sampling of the SWAMP airboat.

**Table S2:** SWAMP airboat field testing site conditions at Star Lake, AB on Oct. 24, 2024.

**Table S3:** SWAMP airboat field testing of pre-operational checks, cruising, missions, and GPS coordinates at Star Lake, AB on Oct. 24, 2024.


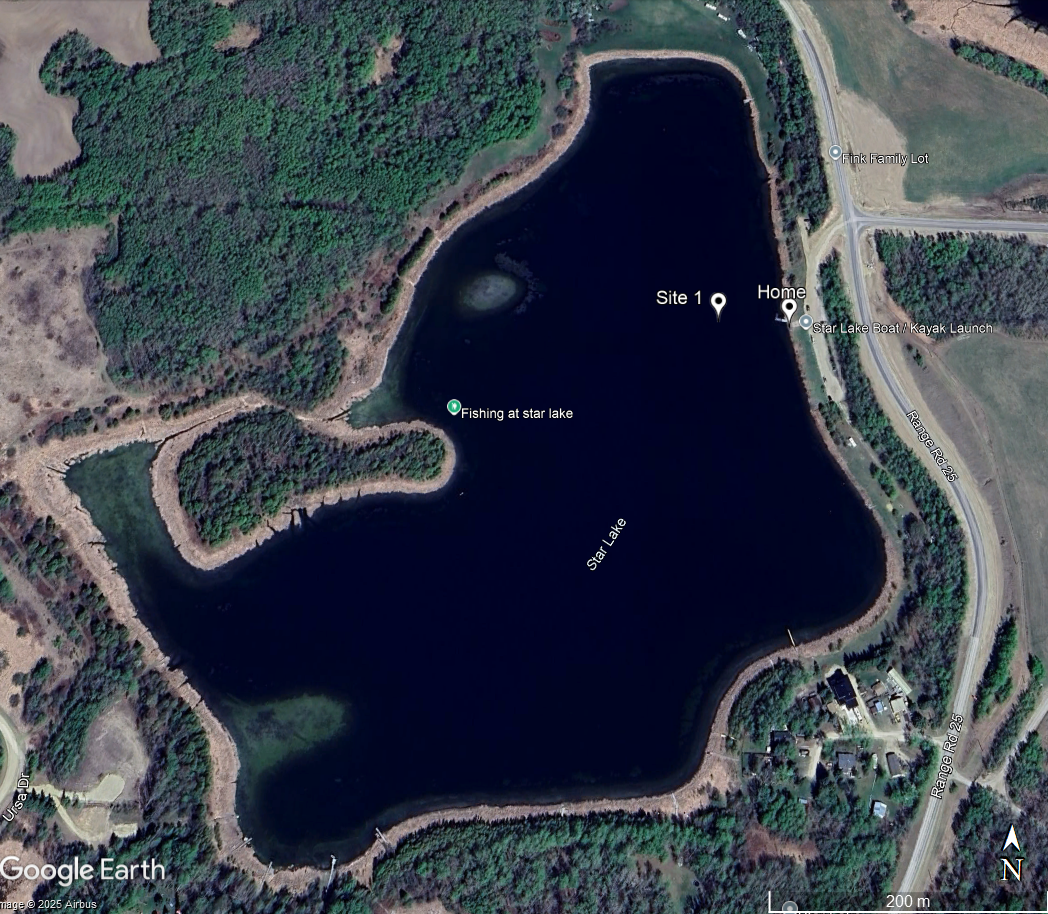


**Figure S1)** SWAMP airboat Home and Site 1 locations for airboat field testing at Star Lake, AB on Oct. 24, 2024 (1).


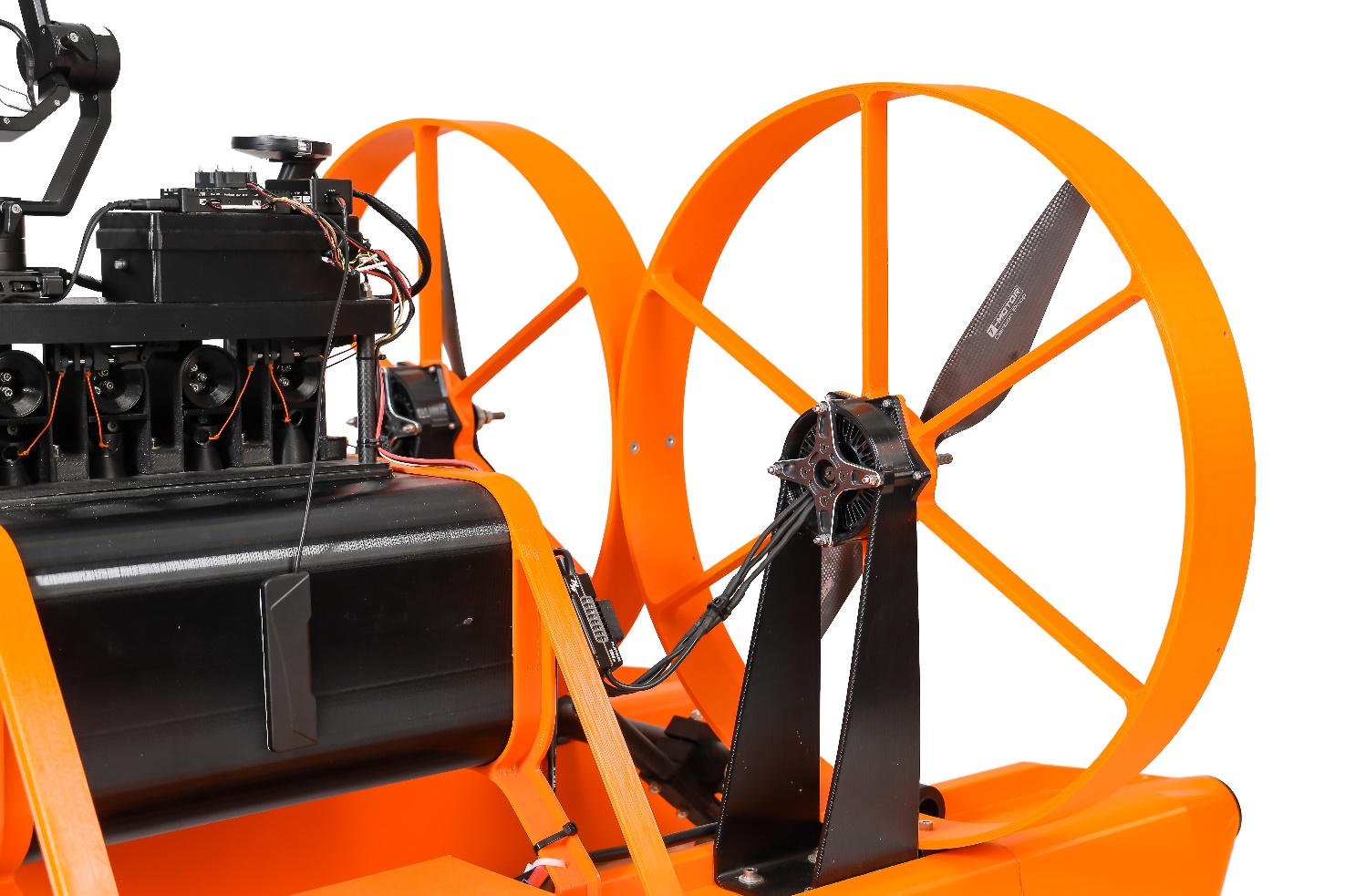


**Figure S2:** SWAMP airboat propellers resting on black 3-D printed PolyMide^TM^ CoPA nylon stands.

**
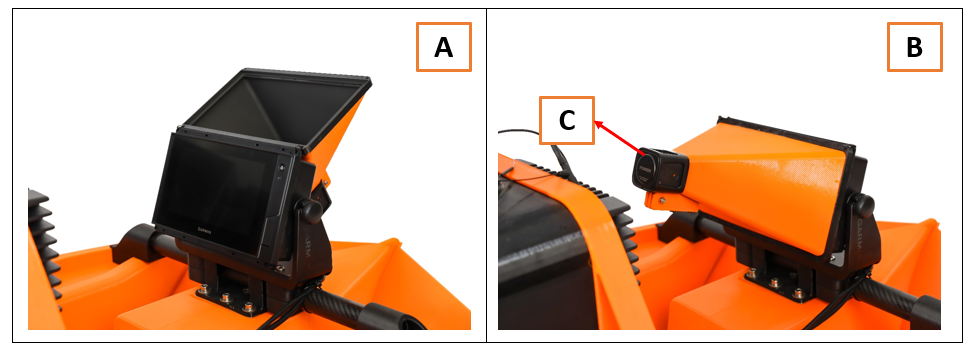
**

**Figure S3**: (A) Garmin Livescope with (B) protective covering that uses sonar mapping to determine ideal sampling locations in a lentic waterbody. A (C) Foxeer BOX 2 camera mounted on the covering feeds Garmin Livescope sonar video back to the Herelink remote.


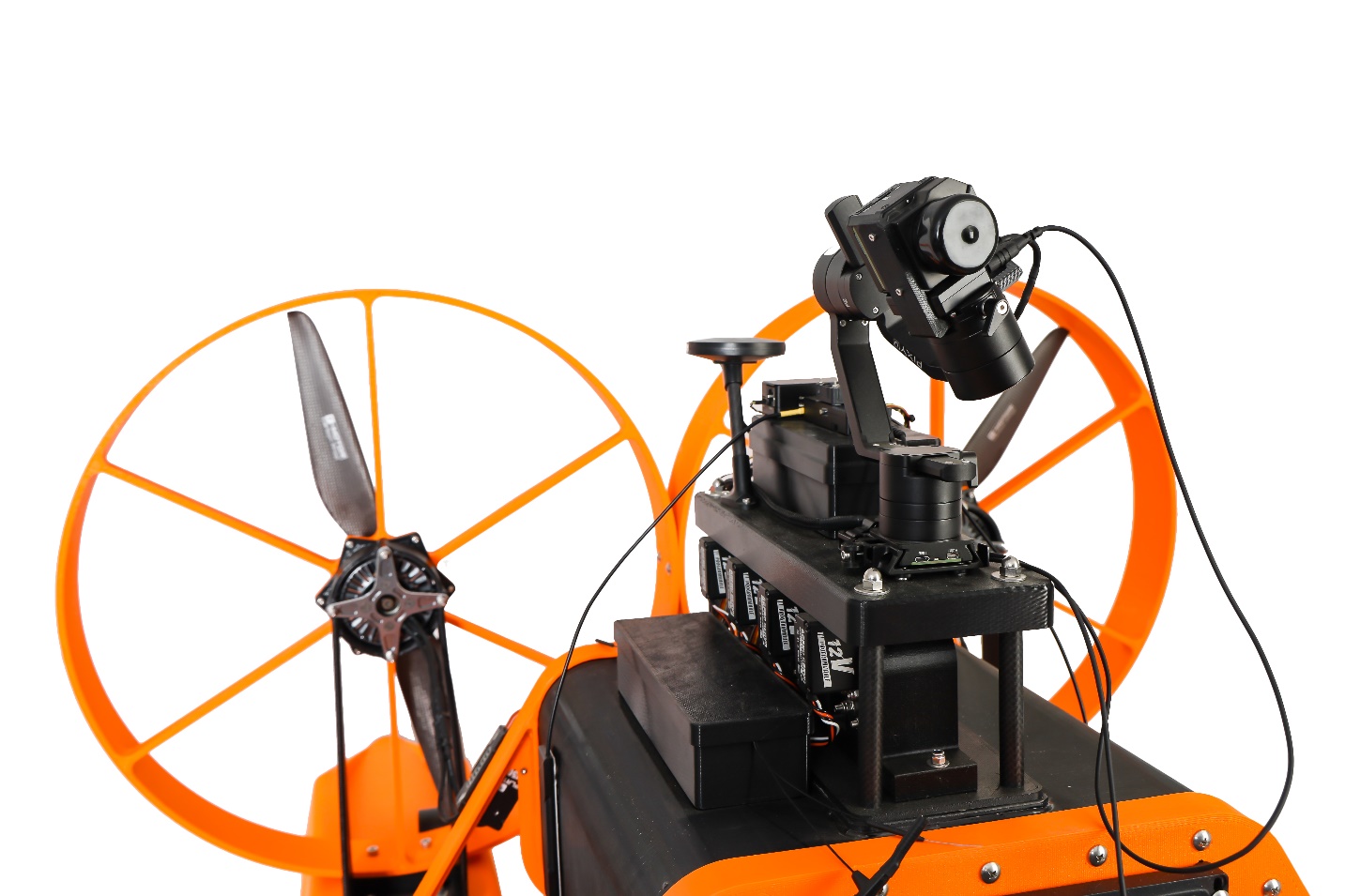


**Figure S4:** The FLIR thermal camera mounted on a Gremsy Gimbal stabilizer provides a 360° view of the site for the operator to view obstacles and map groundwater inputs into the water.

**
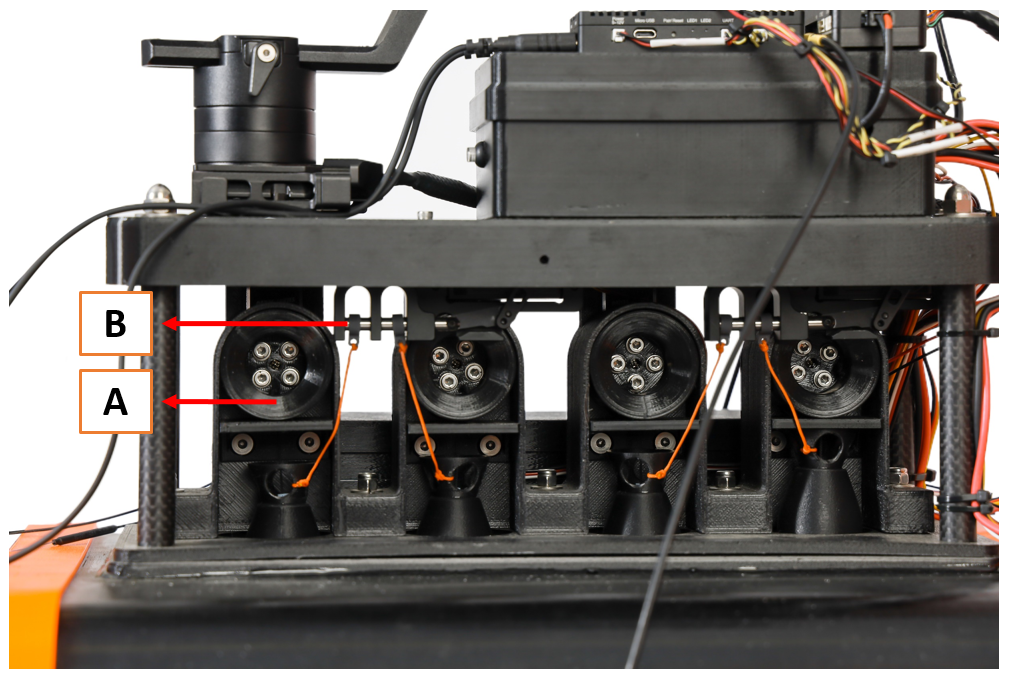
**

**Figure S5:** (A) Four AGFRC programmable motors (winches) that raise and lower 60 mL sampling bottles to desired depths. (B) The teardrop PC carbon weights are released from a solenoid released by the operator.


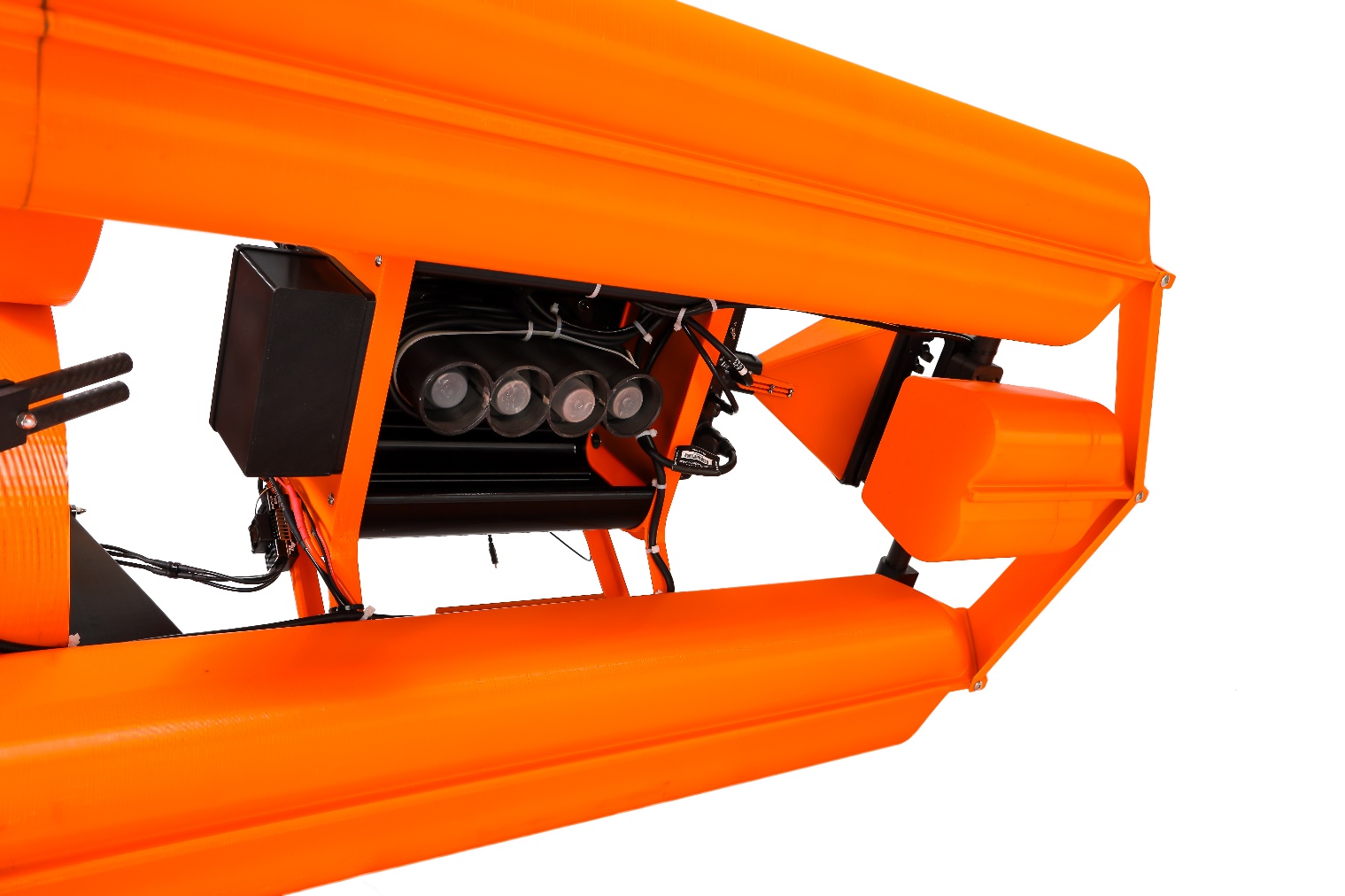


**Figure S6:** The SWAMP airboat displaying the ventral view of the black water sampler with four bottles mounted inside ready for deployment.


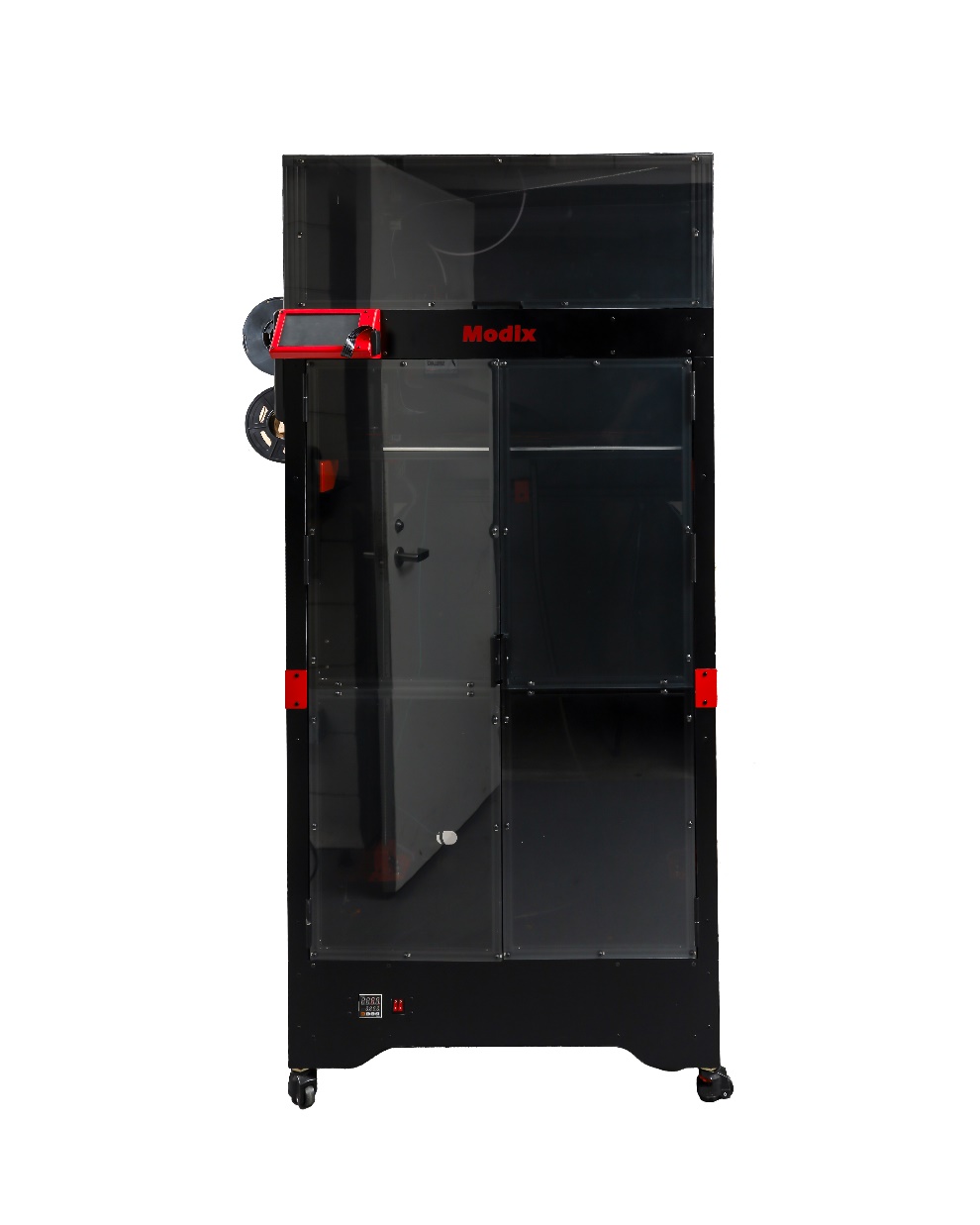


**Figure S7:** Modix 3D printer used to construct all plastic components for the SWAMP airboat.


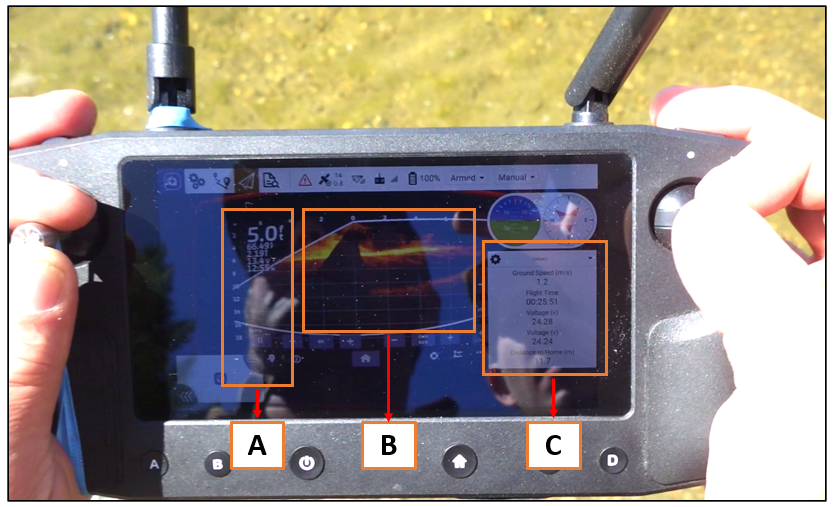


**Figure S8:** Herelink remote demonstrating the (A) y-axis with descending depth (ft), (B) sonar imagery cast by the Garmin Livescope, and (C) display of speed (km/h), flight time (minutes), voltage (v) remaining on batteries, and the distance to Home (m).

**Table S1)** Pre-operational safety checks, mission planning, and test drive steps and actions prior to safe operation and sampling of the SWAMP airboat. *****These functions are optional, and the SWAMP airboat is able to operate and sample without these features.

| 1. **Pre-Operational Safety Checks** | | | | **Action if “Yes”** | **Action if “No”** |
| --- | --- | --- | --- | --- | --- |
| 1 | Site easily accessible | □ Yes | □ No | Proceed to next step | 1. Find suitable access point 2. Change locations |
| 2 | Wind speed ≥ 30 km/h | □ Yes | □ No | Proceed to next step | 1. Change Locations 2. Sample on a different day |
| 3 | Air temperature > 5°C | □ Yes | □ No | Proceed to next step | 1. Change Locations 2. Sample on a different day |
| 4 | Rainy forecast predicted | □ Yes | □ No | Proceed to next step | 1. Change Locations 2. Sample on a different day |
| 5 | 3 batteries charged | □ Yes | □ No | Proceed to next step | 1. Cannot sample |
| 6 | Herelink and FrSKY remotes power on | □ Yes | □ No | Proceed to next step | 1. Troubleshoot 2. Cannot sample |
| 7 | 3 batteries connect to water sampler and propellers | □ Yes | □ No | Proceed to next step | 1. Troubleshoot 2. Cannot sample |
| 8 | Cube Autopilot turned on | □ Yes | □ No | Proceed to next step | 1. Troubleshoot 2. Cannot sample |
| 9 | FLIR thermal camera turned on | □ Yes | □ No | Proceed to next step | 1. Troubleshoot 2. Cannot sample |
| 10 | Garmin Livescope turn from blue to green | □ Yes | □ No | Proceed to next step | 1. Troubleshoot 2. Cannot sample |
| 11 | Emergency safety button located and operable | □ Yes | □ No | Proceed to next step | 1. Troubleshoot 2. Cannot sample |
| 1. **Mission Planning** | | | | **Action if “Yes”** | **Action if “No”** |
| 1 | GPS waypoints selected in ArduPilot Rover and uploaded to airboat | □ Yes | □ No | Proceed to step B3 | 1. Proceed to step B2 |
| 2 | GPS waypoints selected on Herelink remote | □ Yes | □ No | Proceed to next step | 1. Troubleshoot 2. Manually steer airboat to waypoint and record waypoint |
| 3 | Settings configuration performed in Ground Control Station | □ Yes | □ No | Proceed to next step | 1. Cannot sample |
| 1. **Test Drive** | | | | **Action if “Yes”** | **Action if “No”** |
| 1 | Storage bottles removed from bottle weight | □ Yes | □ No | Proceed to next step | 1. Troubleshoot 2. Cannot sample |
| 2 | Acid cleaned transfer bottles screwed onto bottle weight | □ Yes | □ No | Proceed to next step | 1. Troubleshoot 2. Cannot sample |
| 3 | Valve on bottle weight is closed | □ Yes | □ No | Proceed to next step | 1. Troubleshoot 2. Cannot sample |
| 4 | Manual navigation confirmed | □ Yes | □ No | Proceed to next step | 1. Troubleshoot 2. Cannot sample |
| 5 | Automatic navigation confirmed* | □ Yes | □ No | Proceed to next step | 1. Troubleshoot 2. Operate airboat manually |
| 6 | Loiter function confirmed | □ Yes | □ No | Proceed to next step | 1. Troubleshoot 2. Cannot sample |
| 7 | Return to Home function confirmed* | □ Yes | □ No | **Sampling can proceed** | 1. Troubleshoot 2. Return airboat to Home manually |

**Table S2:** SWAMP airboat field testing site conditions and GPS coordinates.

| **Site** | **Date** | **GPS Coordinates** | **Site Condition** | **Description** |
| --- | --- | --- | --- | --- |
| Long Lake | Jun. 21, 2024 | 53°47’42.44”N 116°12’24.47”W | Cloud Cover | No clouds, Sunny |
| Long Lake | Jun. 21, 2024 | 53°47’42.44”N 116°12’24.47”W | Air Temperature (°C) at 12:00 | 24 |
| Star Lake | Oct. 24, 2024 | 53°30’41.35”N 114°16’8.42”W | Cloud Cover | Partially Cloudy |
| Star Lake | Oct. 24, 2024 | 53°30’41.35”N 114°16’8.42”W | Air Temperature (°C) at 17:00 | 7 |
| Star Lake | Oct. 24, 2024 | 53°30’41.35”N 114°16’8.42”W | Air Temperature (°C) at 18:30 | 5 |
| Star Lake | Oct. 24, 2024 | 53°30’41.35”N 114°16’8.42”W | Minimum Wind Speed (km/h) | 1.4 |
| Star Lake | Oct. 24, 2024 | 53°30’41.35”N 114°16’8.42”W | Maximum Wind Speed (km/h) | 10.8 |

**Table S3:** SWAMP airboat field testing of pre-operational checks, cruising, missions, and GPS coordinates at Star Lake, AB on Oct. 24, 2024.

| **Task** | | **Duration (minutes:seconds)** | **Depth (m)** | **Latitude** | **Longitude** |
| --- | --- | --- | --- | --- | --- |
| Pre-Operational Safety Checks | | 25:24 | N/A | 53.5115568 | -114.2693608 |
| Mission 1 | Cruising to Site 1-1 | 01:08 | N/A | N/A | N/A |
|  | Sample Collection Site 1 D1-1 | 01:36 | 1.5 | 53.5115904 | -114.270237 |
|  | Sample Collection Site 1 D2-1 | 01:49 | 3.0 | 53.5115904 | -114.270237 |
|  | Sample Collection Site 1 D3-1 | 01:25 | 4.5 | 53.5115904 | -114.270237 |
| Mission 2 | Cruising to Site 1-2 | 02:23 | N/A | N/A | N/A |
|  | Sample Collection Site 1 D1-2 | 01:09 | 1.5 | 53.5115904 | -114.270237 |
|  | Sample Collection Site 1 D2-2 | 02:10 | 3.0 | 53.5115904 | -114.270237 |
|  | Sample Collection Site 1 D3-2 | 03:42 | 4.5 | 53.5115904 | -114.270237 |
| Mission 3 | Cruising to Site 1-3 | 01:02 | N/A | N/A | N/A |
|  | Sample Collection Site 1 D1-3 | 01:37 | 1.5 | 53.5115904 | -114.270237 |
|  | Sample Collection Site 1 D2-3 | 01:33 | 3.0 | 53.5115904 | -114.270237 |
|  | Sample Collection Site 1 D3-3 | 07:32 | 4.5 | 53.5115904 | -114.270237 |

**References:**

1. Google Earth Pro version 7.3.6.9796. Star Lake, Alberta, 53°30'41.35"N 114°16'8.42"W, (February 22, 2024).
